# Severe Thrombocytopenia in the Post-surgical Context and Administration of Factor VIIII (FVIII)/von Willebrand Factor (VWF) Concentrate in a Patient With von Willebrand Disease Type 2M

**DOI:** 10.7759/cureus.77868

**Published:** 2025-01-23

**Authors:** Daniela Baptista, Lourenço Castro e Sousa, Rita Pardal, Marta Rebelo, Maria Isabel Simões

**Affiliations:** 1 Internal Medicine, Hospital Egas Moniz, Lisbon, PRT; 2 Vascular Surgery, Hospital Egas Moniz, Lisbon, PRT; 3 Hematology, Hospital Egas Moniz, Lisbon, PRT; 4 Critical Care, Hospital Egas Moniz, Lisbon, PRT

**Keywords:** aneurysmectomy, thrombocytopenia, von willebrand disease, von willebrand factor, wilate

## Abstract

Von Willebrand disease (VWD) is the most common inherited bleeding disorder. It can be associated with a life-threatening risk of excessive bleeding in surgical procedures, and may require prophylactic treatment with a combined factor VIIII (FVIII)/von Willebrand factor (VWF) concentrate. Management of these patients may be challenging when trying to achieve the balance between avoiding the risk of haemorrhage and causing a risk of thrombosis with the treatment. We present a complex case of severe thrombocytopenia in a post-surgical setting, in which the timeline suggests a direct relationship between the worsening of platelet count and wilate administrations.

## Introduction

Von Willebrand factor (VWF) is a glycoprotein crucial to primary haemostasis and the intrinsic coagulation cascade as it binds to collagen at sites of vascular injury, mediates platelet adhesion and aggregation through adherence to glycoprotein 1b (GP1b) receptors, and serves as a carrier protein for coagulation factor VIII (FVIII), stabilizing it and thereby increasing its circulating half-life [1,2].

Von Willebrand disease (VWD) is the most common inherited bleeding disorder, which may result from defects in the synthesis, structure, secretion or function of VWF or any combination thereof. VWD is classified into quantitative (types 1 and 3) and qualitative defects (type 2) [2,3]. VWD type 2 is divided into four secondary categories, including type 2M, the one discussed in this case, which includes variants with markedly defective platelet adhesion despite a relatively normal size distribution of VWF multimers [4-6].

Whereas most cases of type 1 VWD can be treated with the synthetic vasopressin analogue desmopressin, patients with type 3 VWD and the majority of those with type 2 require concentrates containing VWF [7].

Wilate is a plasma-derived, albumin-free, double virus inactivated high-purity concentrate of freeze-dried VWF and FVIII developed specifically for the treatment of VWD [8]. It contains VWF and FVIII in a physiological 1:1 activity ratio, which may facilitate simple dosing and monitoring even when repeated dosing is needed [8].

Surgical procedures in patients with VWD can be associated with a life-threatening risk of excessive bleeding and may require prophylactic treatment with a combined VWF/FVIII concentrate [9]. Wilate has proven to be effective and well tolerated by people with all types of von Willebrand disease for surgery, on-demand treatment and prophylaxis [7-12].

This case shows a patient who received prophylactic treatment with wilate due to an elective surgery and developed severe thrombocytopenia of rapid onset.

## Case presentation

We report a case of a 56-year-old Portuguese male patient with type 2M von Willebrand disease admitted to Egas Moniz Hospital for elective surgical correction of an abdominal aortic aneurysm (saccular/false aortic aneurysm) with 67 mm diameter.

He was diagnosed with VWD at 49 years of age and the genetic study showed a missense mutation in the VWF A1 domain (binding to platelet GP1b) in heterozygosity (Mut. in exon 28, c.4120C>T, p.Arg1374Cys). He had a history of occasional gum bleeding without the need for therapeutic intervention and an episode of profuse bleeding following excision of a sebaceous cyst.

In the preoperative analytical assessment, he had the following values: factor VIII, 63% (reference range (RR): 50-150); von Willebrand factor activity, 19% (RR: 50-180); and von Willebrand factor antigen, 52% (RR: 50-180). He was evaluated by the immunohemotherapy department and was given 3000 U of FVIII/VWF concentrate (wilate) + 1 g of tranexamic acid before surgery. He also received another 2000 IU of wilate 12 hours after pre-op administration and one more administration was scheduled for the day after.

The procedure occurred on the 19th of August and consisted of xiphopubic laparotomy and bilateral common femoral exposure, under systemic heparinisation with 5000 U and after clamping the infrarenal abdominal aorta, aneurysmectomy of the saccular aneurysm of the infrarenal abdominal aorta and aortobifemoral interposition of a 14 X 7 mm silver-coated Dacron prosthesis. Biopsies were taken from the aneurysm sac for microbiological and pathological examination. Haemostasis was reviewed. The prosthesis was covered at the abdominal level with a large epiplon flap and the retroperitoneum was closed. The abdominal wall and the bilateral inguinal wall were closed in planes, leaving an aspiration drain by counter incision. Estimated haematological losses were 700 mL, and a total of 4500 mL of fluid therapy was carried out with 4000 mL polyelectrolyte and 500 mL gelofundin.

Given the probable endarteritis found intraoperatively, empirical antibiotics with piperacillin-tazobactam and vancomycin were started.

The patient was transferred to the intensive care unit in the immediate post-operative period, and admitted with invasive mechanical ventilation, under remifentanil and propofol infusion.

In the first hours, he developed thrombocytopenia with a platelet serum value of 39,000x10^6/L (pre-surgery platelet value was 257,000x10^6/L). Due to suspicion of possible heparin-induced thrombocytopenia (HIT), an anti-platelet factor 4 (PF4) test was ordered, with a subsequent negative result. There was also a drop in hemoglobin value to 11.9 g/dL (pre-surgery was 14 g/dL), with no evidence of haemorrhagic dyscrasia, which was assumed in the context of haemodilution. He was given 2000 UI of wilate at the end of the day as previously indicated, and another administration was scheduled for the following day.

Progressive weaning from ventilation and sedation was carried out, and the patient was successfully extubated the same day. Noradrenaline was maintained to keep mean arterial pressure (MAP) at 70-80 mmHg, with the need to titrate the dose up to 0.37 mcg/Kg/min, due to a rise in lactate to 5 mmol/L in the context of distributive shock. He maintained fluid therapy until the following day, normalising the lactate value and stopping vasopressor support.

On the 20th of August, his platelets rose to 53,000 and he was administered 2000 IU of wilate, which had already been scheduled according to indications from our immunohemotherapy colleagues, despite having already normalised values for factor VIII (135%) and von Willebrand factor at 118% (activity) and 206% (antigen).

The following day, there was a further worsening of thrombocytopenia with platelets of 11,000x10^6/L, confirmed by analysis in a thromboexact tube. Factor VIII and von Willebrand factor dosages were adequate at factor VIII, 135% (N); von Willebrand factor activity, 118%; and von Willebrand factor antigen, 206%.

This was discussed with our colleagues in the immunohemotherapy department, who recommended the transfusion of 1 unit of platelets, with an increase in platelet count to 23,000x10^6/L, which was considered to be insufficient. The fact that an autoimmune process could not be ruled out was also taken into consideration, so we decided to start methylprednisolone 1 mg/Kg and to request anti-platelet antibodies, which subsequently came back negative and a weaning from corticotherapy was initiated.

No more wilate administrations were given, and in the following days the patient’s clinical and analytical evolution was favourable, with his platelet count gradually returning to baseline, without any haemorrhagic or thrombotic complications.

There was never evidence of haemolysis or alteration of the clotting times, apart from the day of the surgery due to the administration of 5000 U of unfractionated heparin.

**Figure 1:**
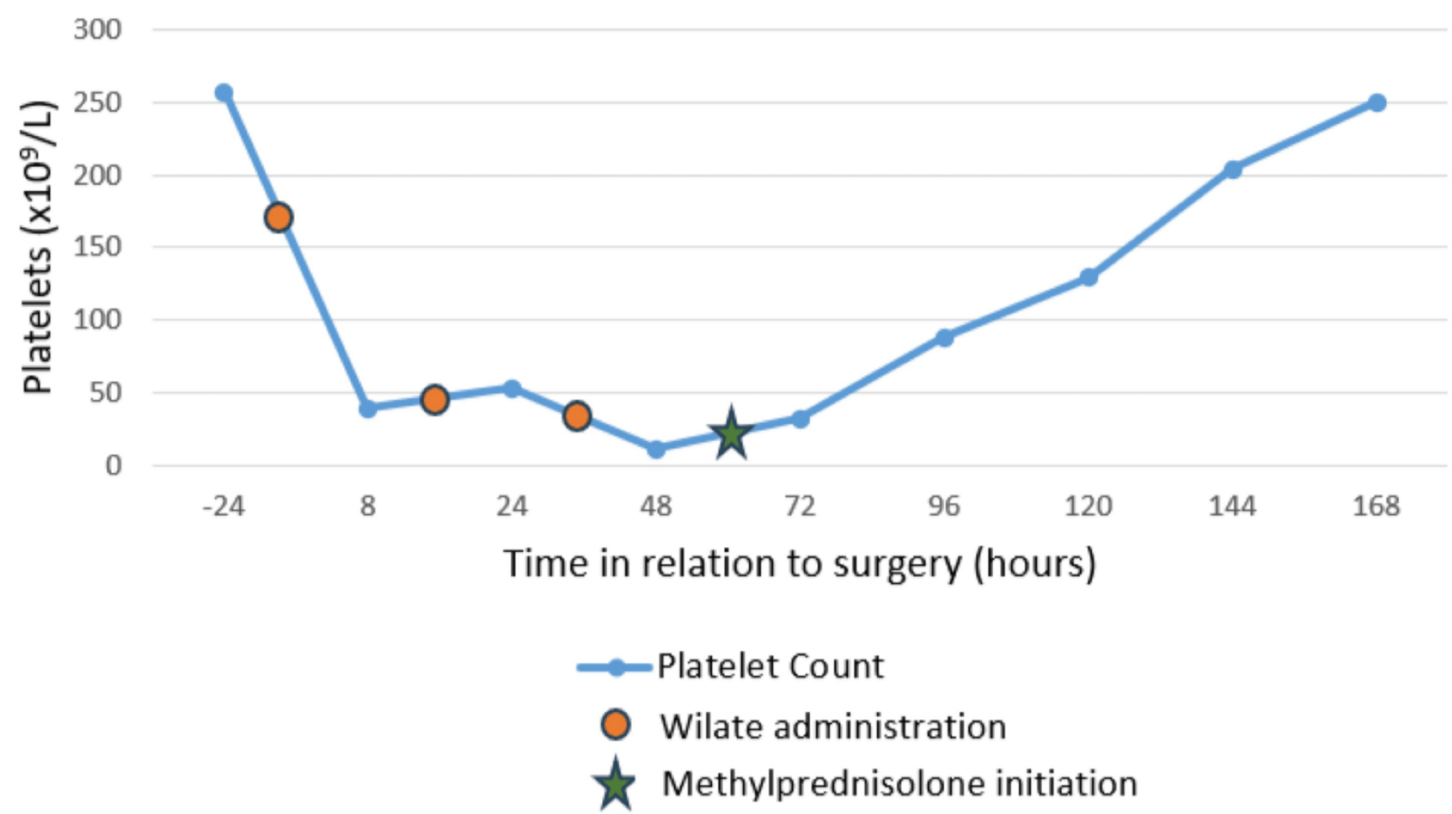
Evolution of thrombocytopenia in relation to time after surgery and wilate administrations

The evolution of blood analysis during the patient’s stay in the ICU and thrombocytopenia in relation to time of surgery and wilate administrations are shown in Table 1 and Figure 1, respectively.

**Table 1:** Analytical Evolution During the Stay in the ICU 1 - Month before surgery; 2 - Day before surgery; 3 - Blood analysis hours after surgery; 4 - Blood analysis at the time of discharge. RR: Reference range.

Date	18/07^1^	16/08^2^	19/08^3^	20/08	21/08	22/08	23/08	26/08^4^
Hemoglobin (g/dL) (RR: 13-17g/dL)	14.9		11.9	9.5	8.9	8.7	8.4	8.2
Hematocrit (RR: 0.406-0.504)	0.451		0.352	0.284	0.269	0.260	0.251	0.233
Platelets (x10^6/L) (RR:150000-400000)	257000		39000	53000	11000	32000	88000	250000
Prothrombin Time (RR: 11-14)		10.6	13.3	13.5	13.2	12.4	12.1	11.4
Activated Partial Thromboplastin Time (RR: 23-40)		37.8	56.3	28.5	26.4	29.7	31.2	30.2
Fibrinogen (RR: 1,5-3,5g/L)		2.70	2.05					3.5
Factor VIII (%) (RR: 50-150)	51	63		135	195	205	188	118
Von Willebrand Factor activity (%) (RR: 50-180)	13	19		118	121	100	65	40
Von Willebrand Factor Antigen (%) (RR: 50-180)	53	52		206	217	200	158	

On the day of discharge, factor VIII and von Willebrand were measured with the following values: factor VIII, 118%; von Willebrand factor activity, 40%.

Given adequate values and following discussion with the vascular surgery team and imunotherapy department, the patient started platelet anti-aggregation therapy without complications on 26 August.

To be noted that during this time, the biopsy from the aneurysm sac identified Staphylococcus epidermidis and the antibiotic regimen was altered according to the antibiotic sensitivity test to flucloxacillin 1 g every four hours. Blood cultures were negative.

Due to favourable evolution and clinical stability, the patient was transferred to the vascular surgery ward.

## Discussion

In the first hours after surgery, heparin-induced thrombocytopenia was considered due to the drastic fall in platelet count. However, the timing was not in favour of this hypothesis, as was the fact that there was no history of previous exposure to heparin, giving a 4T score of 3 (low probability of HIT <5%). The anti-PF4 negative test finally allowed us to exclude this diagnosis.

Iatrogenic component was also taken into consideration; there have been reports of thrombocytopenia due to metamizole, which the patient was taking in the first 48 hours for pain control and later suspended. It is rare and when it occurs, it is usually associated with agranulocytosis and or pancytopenia, unlike this case [13,14].

Thrombocytopenia due to piperacillin-tazobactam has also been reported, however, the patient suspended this antibiotic 72 hours after surgery, when the platelet count was already starting to increase, not favouring this hypothesis [15].

Regarding the infectious context, despite having an infected aneurysm sac, it was removed and the source of infection was controlled. Clinically, the patient never showed signs of sepsis or septic shock. The vasopressor therapy was suspended in the first 24 hours. He remained apyretic, had no organ dysfunction, and no haemorrhagic dyscrasias were detected. Therefore, there wasn't enough evidence to justify that the infectious insult could have motivated such a severe drop in platelets.

As to the immune aetiology, we can't exclude it with certainty, since progressive improvement of the platelet value followed corticosteroid therapy initiation. However, acute immune thrombocytopenia (ITP) usually affects young children, ages two to six years old, and often has a chronic manifestation in adults, where the resolution of thrombocytopenia takes months. Also, ITP does not usually manifest before the second postoperative week because the production of the antibodies responsible for platelet loss usually requires at least five days [16].

Considering the timeline and evolution of this case (Figure 1), we raised the hypothesis that the severe thrombocytopenia was due to consumption secondary to the surgical insult, in a possible context of hyperfunctioning platelets, following factor VIII/factor von Willebrand replacement therapy.

Nonetheless, we recognise that it is difficult to draw conclusions from a single case and that the thrombocytopenia may have occurred solely due to the surgical insult, regardless of the VWD. From what is described in the literature, the only subtype of VWD that leads to thrombocytopenia due to an increase in VWF is type 2B (because of increased ristocetin-induced platelet aggregation), so this condition would be more expected to occur in this subtype [17]. However, the study of VWD was confirmed and the patient in question did in fact have type 2M.

To our knowledge, there haven't been any reports of severe thrombocytopenia following surgery and wilate administration and for that reason we felt it was pertinent to share and raise awareness of this possible effect on any type of von Willebrand disease.

## Conclusions

The administration of wilate was crucial for haemorrhage prophylaxis during the surgical procedure, however it also presented an unexpected challenge by playing a role in the severe post-operative thrombocytopenia.

This case proved to be complex and highlighted the value of collaboration between the surgical medical team, intensive care and immunohemotherapy doctors, and it also showed the importance of close monitoring of patients with type 2M VWD, ideally with everyday measurement of platelet count, factor VIII, von Willebrand factor antigen and activity on the first few days following major surgery.
